# An Exposition of the Signs and Symptoms of Pregnancy, the Period of Human Gestation, and the Signs of Delivery

**Published:** 1837-10

**Authors:** 


### ART. X.

*An Exposition of the Signs and Symptoms of Pregnancy, the Period of Human Gestation, and the Signs of Delivery.* By W. F. MONTGOMERY, A.M. M.D. M.R.I.A., Vice-President and Professor of Midwifery in the King and Queen's College of Physicians in Ireland.—*London*, 1837. 8vo. pp. 344.

THERE are few works the announcement of which we have welcomed with such cordial pleasure as that of the one to which we now call the attention of our readers. The name of its accomplished author has for some time past been enrolled among those which adorn the highest ranks of the profession. He is known not merely as a successful teacher and practitioner, but as the author of a considerable number of valuable essays on subjects connected with his own department of medicine, and more especially for an admirable dissertation on the Signs of Pregnancy and Delivery, published in the "Cyclopædia of Practical Medicine." The reader must not suppose that the present work is merely an enlarged reprint of the above essay, it having been, as the author informs us in his preface, "almost entirely rewritten, with the addition of such new matter as the writer's observations and further experience enabled him to contribute, conjoined with such information from other sources as appeared consonant to truth, and likely to be useful in practice," (p. vi.;) and, in justice to him, we feel bound fully to confirm this observation in every respect.

The work may be considered as being divided under five heads: 1st, the Diagnosis of Pregnancy; 2dly, Investigation of the Uterus and its Appendages after Death; 3dly, on the Period of Human Gestation; 4thly, on the Signs of Delivery; 5thly, on the Spontaneous Amputation of the Fœtal Limbs. In this order we propose to consider it, and promise ourselves the pleasing task of giving a succinct but complete digest of its whole contents. The diagnosis of pregnancy has occupied much of our attention, and we will freely confess that there are few occasions on which we have felt so much painful uncertainty as in cases of this sort where circumstances have combined to render our examination difficult and our conclusions doubtful.

Dr. Montgomery devotes his first Chapter to "General Observations on the State of the Female System during Pregnancy;" and, in pointing out the various sources of derangement to the health at this period, he gives the following correct remarks:

"It has been already noticed that the state of pregnancy is one of increased vascular action, not only in the great organ primarily affected, but generally throughout the system, by which a disposition is created to certain affections indicative of plethora, and best alleviated by venesection or other depleting measures. This natural tendency to redundance during this state is too often cherished and increased, to the great prejudice of the woman, by mismanagement of her diet, neglect of the state of her bowels, and the want of proper and sufficient exercise, all of which mutually react upon each other, each rendering the effect of each still more decidedly injurious. It cannot escape observation that, during gestation, the activity of the alimentary canal is almost always greatly impaired, and hence one strong reason is suggested for greater caution in selecting food of a proper kind, and for restriction in its quantity. Nature, as a safeguard, resorts to vomiting, thereby, as it were, declaring her opinion that there is something superfluous in the system, the evacuation of which is not only beneficial to the parent, but subservient to the welfare of the child, which we know is but too surely threatened when, in the early months, the disposition to vomit suddenly subsides. The popular prejudice on this subject is, that a pregnant woman, having two to feed, ought to swallow a double supply of nutrition; while nature declares the exact contrary, by disposing her to reject a large proportion of what she takes, and making her averse from any of the richer kinds of meat, which at other times she would eat with pleasure. Moreover, experience has shown that the perfection of the fœtus, either as to health or size, depends very little on the quantity of nutriment supplied to the mother during gestation: hence the attempts that have been made to restrain the growth of the child, by diminishing the food of the mother, have not only signally failed in accomplishing the object intended, but the children have been, in some of the trials, unusually large and well-thriven." (P. 9.)

Experience has shown the correctness of this remark: hence we find that, in cases of far-gone phthisis, where the powers of the system appear to have just sufficed to complete the process of gestation, and then sink exhausted, the child is usually remarkably large, stout, and well developed. We have already made this remark in our Review of Professor Kilian's work (No. VI., April, 1837, p. 415,) and may add that phenomena of a similar nature are observed in the vegetable kingdom.

In speaking of the effects produced by an overloaded state of the circulation in pregnancy, the author considers that "inordinate secretion of the liquor amnii," with its consequences, "are evils which will be the more surely entailed if, at the same time, the state of the bowels be neglected." We can scarcely agree with him in this remark, because, in young robust primiparæ, where the circulation so frequently requires reduction by the lancet, we do not observe an unusual distention of the uterus from this cause more frequently than in a very opposite class of patients. He proceeds to give some excellent observations on the advantages of proper exercise, diet, and good air; and points out the great susceptibility of the nervous system to external impressions during pregnancy, and the danger arising from exposure to strong "mental or moral emotions" at this period. "Neither should they (the patients) be permitted, if possible, to see disgusting objects; for, although no injury may be thereby done to the child, their minds are apt to remain much troubled with anticipations of some deformity or disfigurement likely to ensue." Now, in reference to this matter, but without meaning in any way to advocate or countenance either the indiscriminate doctrine of effects produced by the mother's imagination, or the absurd fabrications by which it has been attempted to maintain it, we cannot help thinking it quite consistent with reason and the present state of our knowledge, to believe that a very powerful impression on the mother's mind, or nervous system, may injuriously affect the fœtus; and it will, at least, be always safe and prudent to act on such a presumption, for "although," to use the words of Morgagni, "I do not approve these things, (that is, the absurd stories,) there are cases where it seems to me to be very hard to depart totally and altogether from that opinion which is common to the greatest men." We are very far from wishing to treat this subject lightly or with ridicule: the mysterious changes and derangements of development which are so frequently observed to occur during embryotic and fœtal life, are phenomena which, in the present state of our knowledge, defy our utmost endeavours to explain; but they are not the less deserving of attention, and it is only by a careful investigation that we can ever hope to arrive at a correct knowledge of their nature.

Dr. Montgomery's remarks on the nervous irritability and mental depression which are sometimes so distressing, both to the patient and her attendants, at this period, are well worthy of attention.

"Occasionally, however, the depression assumes a more serious aspect, and the woman is constantly under the influence of a settled and gloomy anticipation of evil, sometimes accompanied with that sort of apathetic indifference which makes her careless of every object that ought naturally to awaken an interest in her feelings; a state which we sometimes observe in fever and other severe disorders, in which it is justly considered a most unfavorable symptom. When this occurs in pregnancy, it will generally be found accompanied by very evident derangements in bodily health: a dull heaviness or aching of the head; a loaded tongue, with bitter taste in the mouth; constant nausea, costiveness, and a foul state of the alvine discharges; with not unfrequently a bilious tinge in the skin, and other symptoms indicating hepatic derangement, together with a quick pulse and a hot dry skin, constitute a group of symptoms likely to be present, and which urgently demand attention for their removal before the time of labour; otherwise serious consequences are to be apprehended." (P. 19.)

There can be no doubt but that the medical man has it frequently in his power to procure much relief for a patient by judicious treatment and by carefully investigating the various sources of irritation on which this state of mind is depending. The conditions of the digestive organs demand here especial attention. The subject is illustrated by numerous interesting cases, which cannot fail to excite attention and impress the various truths more forcibly upon the student's mind.

After describing the various derangements of the health which are apt to affect a patient during pregnancy, the author truly points out the beneficial effects which pregnancy exerts on the female system.

"It appears from experience that women who bear children generally enjoy more even health, and are less disposed to disease, than those who lead a life of celibacy, or who, having married, remain unfruitful. Indeed, I think we have sufficient evidence to justify the belief that pregnancy acts in a great degree as a protection against the reception of disease, and apparently on the common principle that, during the continuance of any one very active operation in the system, it is thereby rendered less liable to be invaded or acted on by another." . . . "I had a patient under my care some years ago, affected with white swelling of the elbow-joint, which had gone to a great length, and was very little benefited by treatment, when, all of a sudden, a very rapid amendment was observed: on questioning the lady, I found that she had reason to think herself about six weeks pregnant, which was the fact. From that time the cure advanced uninterruptedly, so that before the end of her gestation the arm was perfectly well, and has continued so ever since." (P. 25.)

In speaking of the difficulties with which we have so frequently to contend in the diagnosis of pregnancy, Dr. Montgomery justly observes, that there are "few questions which impose on the medical examiner a more delicate duty, or a more trying responsibility, than the determination of the existence or absence of pregnancy, placed before him, as the question generally is, under circumstances by which all its natural difficulties are increased a hundred-fold." (P. 30.)

"The pertinacity and apparent innocence with which pregnancy is denied under such circumstances would be quite incredible, and almost certainly mislead us, were we not taught scepticism from experience; having so often disclosed to us that, in the indignant burst of offended chastity, and the deep asseveration of unspotted purity, we were, after all, to recognize the solemn protestation of a lie." (P. 31.)

With regard to the suppression of the menses as a sign of pregnancy, we fully agree with the author that, although we are "justified in adopting as a general rule that, in healthy women, whose menstruation has been established and continued regular, and who are not nursing, conception is followed by a suppression of the menstrual discharge at the next return of its period," (p. 41;) this symptom by itself merits, nevertheless, but little confidence. The fact that conception may take place before menstruation has made its appearance at all, or before its return after previous labour; and, on the other hand, that this discharge may continue to appear regularly *during* pregnancy, even up to the full time,—and yet without the smallest derangement of health,—are duly noticed by the author, and rendered interesting by cases and by much valuable literature. The name of Dionis, which has not been quoted, might have been advantageously substituted for some two or three of the more modern French obstetric authors, the more so as few have devoted so much attention to this subject as he has done. The remarkable cases of menstruation occurring *only during pregnancy*, as recorded by Deventer, Dewees, &c., are also noticed. The appearance of the catamenia *during* pregnancy, although opposed by Dr. Denman, and also very recently by Dr. Hamilton, has been noticed, nevertheless, by all the eminent authorities of former times; more especially by Dionis, from whom our author will find that the passage which he has quoted from Gardien is more or less taken. Dr. Hamilton's attempt to "throw discredit on all the cases of non-suppression during pregnancy" is futile. The observations of Dr. Dewees on this subject would of themselves be quite sufficient to neutralize it, even if we had no other authority to refer to. The correctness of this fact, however, has been maintained and confirmed by authors of the highest authority, both ancient and modern, and it is one which has so frequently occurred under our own notice that we have long since ceased to regard it as a circumstance of any rarity.

Dr. Montgomery's fourth Chapter is devoted to a consideration of the Mammary Sympathies, the Characters of the Areola, &c. To this subject, we are aware, that he has directed much attention. His observations in the "Cyclopædia of Practical Medicine" bear the marks of much observation and experience; and, in the present work, where the extent of his remarks was not subjected to the same unavoidable limitation of space as in the Cyclopædia, he has been enabled to bring forward a larger mass of information, and consequently render it much fuller than before. He has, moreover, illustrated his descriptions of the areola at different months of pregnancy by a series of very beautifully coloured lithographic engravings, from drawings which, we are told, have been superintended by Dr. Carswell, whose master-hand in this respect has long been acknowledged. That the changes of the areola have not only excited attention from the most eminent men of the last century, but were considered by them as valuable evidences of pregnancy, is proved by the remarks of Smellie, W. Hunter, and Rœderer. The description which the last-mentioned author has given of these changes is celebrated as a specimen of accurate description, and ought to be familiar to every one who is desirous of understanding this important piece of diagnosis.*

"I cannot say positively," observes Dr. Montgomery, "what may be the earliest period at which this change can be observed, but I have recognized it fully at the end of the second month, at which time the alteration in colour is by no means the circumstance most observable, but the puffy turgescence, (though as yet slight,) not alone of the nipple, but of the whole of the surrounding disk, and the development of the little glandular follicles, are the objects to which we should principally direct our attention; the colour at this period being in general little more than a deeper shade of rose or flesh colour, slightly tinged occasionally with a yellowish or light brownish hue. During the progress of the next two months, the changes in the areola are in general perfected, or nearly so, and then it presents the following characters:—A circle around the nipple, whose colour varies in intensity according to the particular complexion of the individual, being usually much darker in persons with black hair, dark eyes, and sallow skin, than in those of fair hair, light-coloured eyes, and delicate complexion. The extent of this circle varies in diameter from an inch to an inch and a half, and increases in most persons as pregnancy, as does also the depth of colour." (P. 60.)

"In the centre of the coloured circle, the nipple is observed partaking of the altered colour of the part, and appearing turgid and prominent; while the surface of the areola, especially that part of it which lies more immediately around the base of the nipple, is studded over and rendered unequal by the prominence of the glandular follicles, which, varying in number from twelve to twenty, project from the sixteenth to the eighth of an inch; and, lastly, the integument covering the part appears turgescent, softer, and more moist than that which surrounds it, while on both there are to be observed at this period, especially in women of dark hair and eyes, numerous round spots or small mottled patches, of a whitish colour, scattered over the outer part of the areola, and, for about an inch or more all round, presenting an appearance as if the colour had been discharged by a shower of drops falling on the part. I have not seen this appearance earlier than the fifth

* We quote the passage in a note at length, in order that our younger professional brethren may have the opportunity of reading it. "Menstruorum suppressionem mammarum tumor insequitur, quocirca mammæ crescunt, replentur, dolent interdum, indurescunt; venæ earum ceruleo colore conspicuæ redduntur, crassescit papilla, inflata videtur, color ejusdem fit obscurior, simili colore distinguitur discus ambiens qui in latitudinem majorem expanditur, parvisque eminentiis, quasi totidem papillulis, tegitur."—*El. Artis Obstet.* p. 46 —Rev.

month, but, towards the end of pregnancy, it is very remarkable, and constitutes a strikingly distinctive character exclusively resulting from pregnancy. The breasts themselves are at the same time generally full and firm, at least more so than was natural to the person previously, and venous trunks of considerable size are perceived ramifying over their surface, and sending branches towards the disk of the areola, which several of them traverse. Along with these vessels, the breasts not unfrequently exhibit, about the sixth month and afterwards, a number of shining, whitish, almost silvery lines, like cracks: these are most perceptible in women who, having had before conception very little mammary development, have the breasts much and quickly enlarged after becoming pregnant." (P. 61.)

So much for the "affirmative part of the question," as the author calls it: let us now reverse the page, and observe the other side.

"In the first place, then, pregnancy may exist, and the areola remain deficient in at least one of its usually essential characters; and that the one too generally supposed to be its most important distinctive mark,—namely, the colour. The writer has seen several well-marked instances of this: one in a lady of very fair skin, blue eyes, and light hair; the other in a lady of fair skin, but with black hair and very dark brown eyes; in both the colour of the areola was so slight as hardly to differ from that of the surrounding skin, and certainly was less distinct than I have frequently seen it in the virgin, but all the other characteristic changes enumerated were well developed in both." . . . . "In some, the mammary sympathies are almost entirely wanting, or at most very feebly exerted, even though gestation should be proceeding healthily; and it should be added that, even when there is no such deficiency in the mammary changes and areola, should the fœtus be blighted, the characters of the latter will soon decline and fade away." (P. 63.)

The author has not sufficiently noticed the changes of the areola occasionally produced by organic disease of the uterus or by dysmenorrhœa, (changes which simulate those of pregnancy exceedingly;) nor do we know by what characters an areola arising from either of these causes can be distinguished from that of actual pregnancy during the early months. The inflated appearance of the integument about the papilla, and the mottled colour of the skin at the edge of the disc, during the latter half of pregnancy, have seemed to us to be the most uniform in their appearance, and those which are most peculiar to the gravid state. From our own experience, we are disposed to consider the appearance of milk in the breasts, in cases of uterine disease or irritation, to be of much more common occurrence than the author appears to suppose, and agree with him in placing no confidence in it as a sign of pregnancy.

Dr. Montgomery's views respecting Quickening (which forms the subject of the fifth Chapter,) are very just.

"I wish," says he, "in the first place, to observe, that I use the word quickening reluctantly, and only in compliance with a long-established usage, because, in its literal and proper meaning, it was adopted from the old and barbarous idea that, at a certain period of gestation, life was suddenly infused into the fœtus; an error which the continued use of the term is obviously calculated to foster and prolong. I would, then, be understood as meaning by it no more than the first sensation experienced by the mother of the life of the child within her womb, and not that the child then becomes for the first time endowed with life; which is, however, the notion still generally prevalent in society." (P. 75.) . . . "It is perfectly monstrous and absurd to suppose for a moment that the fœtus does not enjoy vitality from the first moment of its existence, and of course long before the sensation of quickening is felt by the mother; and, if it be asked why no indications of life are given before the time at which quickening generally takes place, the obvious answer is, that the absence of any consciousness, on the part of the mother, relative to the motions of the child, is no proof whatever that such motions do not exist." (P. 77.)

In these points we fully agree with the author in attaching no importance to quickening in the diagnosis of pregnancy. The movements of the child may be influenced by so many causes, and the sensation of it by the mother so easily simulated, that we seldom take the trouble of even questioning a patient upon this subject; feeling justified, from ample experience, that the absence or presence of the sensations of the child's movements to the mother, during the early months of pregnancy, is a symptom on which not the smallest confidence can be placed. "Occasionally," says Dr. M., "circumstances, whose influence it would be very difficult, if not impossible, to explain, appear to have the power of suspending fœtal motion, without, however, inflicting any injury on the child," (p. 81;) and he illustrates this by two interesting cases, to which we could have added others from our own practice. It is a well-known fact among practitioners, that, in patients in whom there is a great disposition to premature expulsion, and in whom much care is requisite during the whole period to prevent it, the movements of the child will suddenly cease on the appearance of any threatening symptoms, and will return in a few days upon their removal; and that this will sometimes occur several times during the same pregnancy.

The entire absence of the child's movements during the whole period of utero-gestation, must not be looked upon as indicating the non-existence of pregnancy; nor can the entire cessation of the child's movements, where they have been distinctly felt at an earlier period, be considered as a sign of its death. Dr. Montgomery quotes some cases where no movement was felt at all during pregnancy; and a similar one occurred to our notice only a few days before writing these observations. A healthy young woman requested our opinion as to whether she was pregnant, and, if so, whether the child was alive; for, during the whole period of her supposed pregnancy, she had not felt the slightest movements of the child. We could not succeed in feeling any, after careful and patient examination of the abdominal parietes, but, on applying the stethoscope, we instantly recognized the uterine souffle; and, after shifting the instrument a few times, we detected the sound of the fœtal heart.

The contents of Chapter vi. are comprised under the heads of "Enlargement of the Abdomen and State of the Umbilicus." To our surprise, the author has adopted the old, and *we* believe erroneous view, that, during the first two months of pregnancy, the uterus actually descends lower into the cavity of the pelvis. By this he explains the flatness of the abdomen at this period; although, in the next chapter, where he speaks of the os and cervix uteri, he does not attempt to give a similar explanation to the well-known fact, that, during the second month, the os uteri is more easily reached than at any other period of pregnancy. The circumstance is indeed mentioned, but no further notice taken of it. The os uteri being low in the pelvis does not, however, arise from the descent of the uterus itself, but simply, as Madame La Chapelle has correctly shown, from its having increased in size, and its fundus not yet having ascended out of the pelvis. We have also reason to believe that the feeling of dragging from the umbilicus, and flatness of the abdomen, does not arise from any descent of the uterus, but from its fundus, which has now become more heavy, gravitating backwards, and thus inclining towards the posterior walls of the pelvis. So long as the uterus preserves the direction peculiar to the virgin state,—viz. with the fundus somewhat inclined backwards, and the os uteri rather forwards,—the small intestines will continue to rest upon it, and for a while prevent its rising, (a period at which the liability to retroversion is known to be greatest;) but, as it increases in size, the broad ligament necessarily became shorter, being gradually more and more expanded over the increasing uterus; the intestines, confined by the mesentery to the spine, soon slip down behind the fundus, which, now freed from its superincumbent pressure, rises through the brim of the pelvis, in a direction upwards and forwards.

In giving directions for conducting an examination of the abdomen, we regret that Dr. M. has not quoted at length the precise and very excellent rules which Rœderer has given upon this subject, or the translation of them in the admirable work of W. J. Schmitt, with which he has shown himself so well acquainted: the directions for making the patient breathe deeply, and for seizing the opportunity of pressing the hand upon the abdomen during expiration, would not have been omitted. We have ourselves quoted the passage alluded to in our Review of Prof. Hohl's work, No. I. p. 108.

With regard to the changes which the umbilicus undergoes, there can be no doubt that, generally speaking, the depression of which it consists begins to diminish about the fifth month; that the folds of skin which form the future umbilicus disappear during the sixth month, and that, after the seventh, it begins to project from the surface of the abdomen; still we can by no means agree with the following remark:

"I do not know of any single instance in which the gravid uterus had acquired such a size without elevating the umbilicus; so that, in any case in which pregnancy is supposed to be advanced to the seventh or eighth month, if we find the umbilicus depressed and the belly flat, it will prove certainly that gestation has not advanced to such a period; although it will not be, as asserted by Dr. Gooch, decisive evidence against the existence of pregnancy, which may be present, but not sufficiently advanced to effect the change, or the uterine development may have been arrested by the death of the fœtus." (P. 98.)

In answer to this quotation, we can only state that we have seen the umbilicus without the slightest diminution in the degree of its depression or in the state of its cuticular folds, only a few days before delivery at the full term.

In describing the Os and Cervix Uteri, in the unimpregnated state, Dr. M. remarks:

"In the virgin and unimpregnated condition of the uterus, its mouth and the lower section of its neck, when examined by the finger introduced into the vagina, can be felt projecting into that cavity from a quarter to half an inch. The part so projecting feels remarkably firm, is slightly tapering or conical in form, and about as large as the end of a man's thumb, having in its termination in the vagina a transverse opening, whose lips or margins feel firm and well defined." (P. 99.)

With this description we are perfectly satisfied, but cannot agree with him where he goes on to state as follows:

"Sometimes the os uteri differs very considerably from this description, being almost imperceptible from its diminutive size, and perfectly circular; and it is not very rare to find it opening at once from the upper extremity of the vagina, without any projection of the cervix uteri into that canal, which to the finger seems to taper gradually to a point, and there terminate in the orifice of the womb, the margins of which are very indistinctly felt. This form of the part is, I believe, in most cases, produced by childbearing: at least, I have very seldom met with it except in women who have had a family." (P. 100.)

We cannot call to mind a single instance of *healthy* os uteri opening at once, without any projection of cervix from the upper extremity of a vagina, which gradually diminished in caliber, as just quoted: that we have felt this state frequently, we readily allow; but it has been connected either with atrophy of the uterus, congenital malformation, or diseased growth. Still less can we agree with the author's last observation,—viz. that of its being produced by childbearing,—since a very contrary effect is known to be produced; the cervix becoming more bulky, and the lips of the os uteri thicker and larger.

In the impregnated state, "the cervix is felt fuller, rounder, and softer, or more springy and elastic, under the point of the finger; and the same alterations having taken place in the labia of the os uteri, this part communicates a corresponding difference in the sensation received by the finger of the examiner: the margins of the orifice feel tumid, but softer and much less distinct, having lost the well-defined edge which, in the unimpregnated organ, is natural to them, and acquired in its stead a peculiar lubricity, in consequence of the increased secretion from the muciparous glandulæ in that situation; while the orifice itself, instead of seeming transverse, *feels* as if it were circular, because it has become more yielding, and admits the tip of the finger more easily and to a greater depth than in its former state." (P. 101.) We look upon the soft cushiony feel which the cervix and os uteri communicate, as a very important change connected with pregnancy, which has more than once assisted us in detecting its presence when complicated with disease; but we regret to be compelled to again express our dissent from the author's last observation. The os uteri, in the primipara, not merely becomes circular during the second month, but is closed by the plug of tough gelatinous substance which is secreted by the glands of the cervix; so that it rather feels like a circular dimple at the end of the cervix, and therefore will be evidently incapable of admitting the tip of the finger *at all.* Still less can we allow the correctness of his remark in the following page: "We can now, also," (alluding, we presume, to the fourth or fifth month,) "introduce the finger with great ease to a considerable depth into the cavity of the cervix, owing to the very yielding condition of the labia of the os uteri." (P. 102.) That the os tincæ or os uteri externum is found more or less open during the latter months in multiparæ, we do not deny; and we occasionally feel it somewhat open in primiparæ, where the patient has been much weakened by leucorrhœa. Nor does it, in our opinion, during the last weeks of pregnancy, feel like "a mere rugous opening in the mucous membrane at the upper part of the vagina:" if the patient be pregnant for the first time, it feels like a soft circular dimple in the inferior and posterior surface of the globe which the uterus now forms. There is no rugosity if, on the other hand, she be a multipara; there will be, it is true, an opening, the edges of which are uneven, irregular, and hard in places, from the cicatrices of former labours, but still it will not feel like an opening "in the upper part of the vagina," because now there will be a portion of the cervix still remaining, which will continue so up to the very commencement of labour. In many cases, where the patient has borne many children, the cervix at the end of pregnancy is more bulky than in the unimpregnated state, being nearly as long and much thicker. Before quitting this subject we have one more quotation to make and to comment upon:

"Nothing is more certain than that there is great variety in this respect" (the length of the cervix,) "in different individuals, some women having the cervix double as long as others; owing to which, and also to the fact that this part, though of ordinary length and healthy, yields much more slowly in some than in others, it happens that more of it will be found undilated in one woman at the eighth month than in another at the sixth; this will be, *cæteris paribus*, most likely to happen in first pregnancies, and hence it is that we not unfrequently find a portion of the cervix amounting to nearly a quarter of an inch undilated, and projecting at the commencement of labour, while at other times the whole cervix is obliterated, and the os uteri considerably opened, one, two, and even three weeks before delivery." (P. 105.)

Dr. Montgomery has entirely omitted to point out the difference between the os uteri of a primipara, and one who has borne children, a diagnosis of great importance, without an accurate knowledge of which we are unavoidably exposed to many apparent contradictions, which create much confusion. To inattention to this point we can alone attribute the assertion just quoted, viz. that the cervix, in first pregnancies, is not unfrequently found projecting nearly a quarter of an inch in the vagina at the commencement of labour, a statement which, in primiparæ, at the full term of utero-gestation, we must strongly dissent from.

The variations in the size of the uterus, during the different months of gestation, are correctly stated; but they are so well known as to require no comment on our part.

We cannot correctly understand why the examination, per vaginam, in the upright posture, is such "a very objectionable mode of proceeding" as the author considers it to be. Wherever we expect any difficulty in forming our diagnosis, especially where, from the period of pregnancy, or other causes, we wish to take advantage of the weight of the organ as much as possible, we hold it to be very important to examine in this position. The rules for conducting this mode of examination we have mentioned in our first number, p. 105.

The observations on the "Application of Auscultation" are good, although rather meager, and contain little or no original information. An apparent inconsistency strikes us at p. 124, where the author says, "We have the very highest authority for believing that the formation of a correct judgment by their means" (sounds,) "requires more care, and is beset with greater difficulties, than are found in investigating all the diseases of the chest." He then observes, three lines further on, "to make this examination it is by no means necessary that we should be practised stethoscopists, or even use the stethoscope at all, since the naked ear will detect the sounds sought for with perfect accuracy, but the use of the tube is for many reasons preferable." (P. 124.) We hold auscultation to be of *great* importance in the diagnosis of pregnancy. In the words of a reviewer of the author's excellent article on this subject, in the Cyclopædia of Practical Medicine, we may truly say, "When all the ordinary symptoms of pregnancy are absent, or so muffled and obscured as to afford scope only for conjecture, if the fœtal pulse can be heard but once unequivocally, the nature of the case is obvious beyond cavil; the auscultator need not heed the discordant opinions of others, for what more can he desire than to have held converse, as it were, with the very being whose existence is disputed."

We fully agree with the author in rejecting the modes of detecting pregnancy recommended by M. Nauche, Professor Kluge, &c. &c., the one being the introduction of a species of auscultating tube, called metroscope, into the vagina, up to the os uteri, the other the inspection of the inner surface of the vagina, to ascertain the presence of a bluish tinge, which is considered by the latter author and M. Jacquereau of Paris as a proof of pregnancy,—modes of examination which, even putting aside the outrage upon the patient's feelings, have been proved to be very far from infallible.

Chapter ix. contains the examination of substances expelled from the uterus, connected with or simulating pregnancy. In speaking of the decidua which covers an early ovum, Dr. Montgomery has pointed out some peculiarities which, in justice to him, we will give in his own words:—

"Repeated examinations have shown me that there are on the external surface of the decidua vera, a great number of small cup-like elevations, having the appearance of little bags, the bottom of which are attached to, or imbedded in, its substance; they then expand, or belly out a little, and again grow smaller towards their outer, or uterine end, which, in by far the greater number of them, is an open mouth when separated from the uterus; how it may be while they are adherent, I cannot at present say. Some of them, which I have found more deeply imbedded in the decidua, were completely closed sacs. Their form is circular, or very nearly so, they vary in diameter from a twelfth to a sixth of an inch, and project about the twelfth of an inch from the surface of the decidua. Altogether, they give one the idea of miniature representations of the suckers of the cuttle-fish." (P. 133.)

To these observations Dr. Montgomery has appended the following note, which we have read with great interest:

"I confess," says he, "I am not prepared (nor, indeed, is this the place,) to offer any very decided opinion as to the precise nature or use of these decidual cotyledons, for to that name their form, as well as their situation, appears strictly to entitle them; but, from having, on more than one occasion, observed within their cavity a milky or chylous fluid, I am disposed to consider them reservoirs for nutrient fluids, separated from the maternal blood, to be thence absorbed for the support and development of the ovum." (P. 134.)

Although these remarks are, perhaps, as the author admits, rather foreign to the present subject, still they are too interesting and important to have allowed us to pass them unnoticed. The fact of a milky fluid being found in the venous absorbing radicles of the chorion, at an early period of pregnancy, has been noticed by several authors of the last century, and points out one very important means of fœtal nutrition. Respecting the source of this fluid little or nothing, as far as we are aware, had been known, so that we may look upon these observations of the author as a valuable contribution to this branch of human physiology.

Chapter x. commences with a very good account of the various idiosyncrasies and anomalous symptoms which are observed in some individuals during pregnancy, and occasionally are very useful in assisting our diagnosis of this condition. We could have mentioned a few more besides those which the author has enumerated, if it had been necessary, for they are almost infinite in point of number and variety: Dr. Montgomery has shown extensive reading upon this subject, and has given a considerable number of interesting cases. We are surprised that he has entirely passed over in silence the observations of Professor Hohl on this subject; they are, it is true, exceedingly minute, and in some places uselessly so, but still they contain much curious information on this subject.

The author dissents from the generally entertained opinion "that the blood of pregnant women *always* presents the buffy coat, and other characters of inflammation," and the result of our own observations would lead us, in great measure, to agree with him, inasmuch as we have not unfrequently met with an entire absence of this appearance in pregnant women; on the other hand, we have repeatedly seen the buffy coat well marked, and without the presence of inflammatary action. We are inclined to believe that this appearance exists chiefly during the very early months of pregnancy, at a period when the "formative nisus" is in full energy, and where a copious supply of plastic fibrinous material is required for the development of the embryo; the author, in fact, comes to this conclusion a little further on, where he says, "I must add that, at those periods, that is, up to the third or fourth months, the blood will be found, in the great majority of instances, presenting the modified characters of inflammation; especially in those whose pulse is much accelerated, or who are of a full habit, or sanguine temperament: but if this be asserted as a general rule, applying to every period of gestation, the exceptions will be found very numerous indeed." (P. 156.)

The peculiar milky turbid appearance which is observed in the urine of pregnant females, which has been lately noticed by M. Nauche, and originally observed, it would appear, by Savonarola, in 1486, is also treated in the same cautious and rational manner:

"I have myself tried it (says Dr. M.,) in several instances, and the result of my trials has been this—in some instances no opinion could be formed as to whether the peculiar deposit existed or not, on account of the deep colour and turbid condition of the urine; but, in the cases in which the fluid was clear and pregnancy existing, the peculiar deposit was observed in every instance. Its appearance would be best described by saying that it looks as if a little milk had been thrown into the urine, and, having sunk through it, had partly reached the bottom, while a part remained suspended and floating through the lower part of the fluid, in the form of a whitish semi-transparent filmy cloud. In some cases in which pregnancy was suspected, but did not exist, no such deposit was observed: but it is superfluous to say that there is such a host of accidental causes, capable of altering the condition of the urine, as ought to make us very cautious indeed how we venture to attach credit to a symptom so equivocal." (P. 157.)

The observations on pregnancy at a very early or advanced age, with which the eleventh Chapter commences, although perhaps not exactly belonging to the professed object of the work, are, nevertheless, exceedingly interesting, and well worth perusal. We have ourselves delivered a patient at the age of thirteen, and superintended that of another at the age of fourteen: in both cases the child was born alive. In the first case labour came on at the eighth month, and was completed during severe puerperal convulsions; there was but little trace of mammary development at first, and the patient's mother (herself a very young woman, and nursing an infant,) supplied her grandchild until the secretion of milk was duly established.

The observations on *spurious pregnancy* are very judicious, and bear reference to a condition of the female system which is by no means an unfrequent occurrence, and which but too often subjects the patient to a most disheartening discrepancy of opinion, and injurious variety of medical treatment:

"It is necessary," says Dr. Montgomery, "to notice here a condition of the female system of a remarkable kind, most frequently observed about the turn of life when the catamenia, becoming irregular, previous to their final cessation, are suppressed for a few periods, and at the same time, the stomach being out of order, nausea or vomiting is experienced, the breasts enlarge, become sensible, or even slightly painful, and sometimes a serous or sero-lactescent fluid exudes from the nipples and orifices of the areolar tubercles; the abdomen grows fuller and more prominent, especially in women of full habit, and constitutionally disposed to *embonpoint;* and the abdominal enlargement progressively increases, partly from disposition of fat in the integuments, and in the omentum, but still more from distension of the intestines by flatus, which, passing from one part to another, communicates a sensation like that produced by the motion of a fœtus; the nervous system is generally much disturbed, and the woman feels convinced that she is pregnant; an idea which, at the time of life alluded to, is cherished by the sex with extraordinary devotion, and relinquished with proportionate reluctance; and not unfrequently at the end of the supposed gestation the delusion is rendered complete, and almost assumes the character of reality, by the occurrence of periodical pains, strongly resembling those of labour." (P. 169.)

Similar observations have been made by W. J. Schmitt of Vienna, who quotes at length a very remarkable case of this sort, described by Klein of Stuttgard, to which Dr. Montgomery also alludes.

The twelfth Chapter commences with the investigation after death, and contains a minute account of the changes which are produced in the ovaries by conception. The consideration of this subject is one of great importance, and still presents a field for much discovery. We are aware that the author has devoted much time and attention to the practical investigation of this branch of Physiology; his sound and excellent observations on this subject have long been highly appreciated by us, and the present remarks have been rendered still more interesting by numerous coloured engravings:

"The vesicle, after impregnation, may really be said to become, with regard to the contained germ, a sort of little temporary uterus, lined with a serous membrane, covered externally by another, and having interposed between them the fleshy, or glandular structure of the corpus luteum, through which blood-vessels ramify, and exhale through the lining membrane a serous fluid for the support of the early ovum, which as yet lives by imbibition." . . . "It will appear very obviously, from the above description, that I believe the corpus luteum to be surrounded, externally, by the outer membrane of the Graafian vesicle, while its cavity is lined by the inner membrane of this vesicle; the corpus luteum being, in fact, inclosed between these two membranes, and its substance pervaded by the small vessels passing from the outer to the inner." (P. 217.)

Dr. Montgomery candidly states that he has taken a different view of the subject to that entertained by Professor Baer, in the accuracy of whose views and researches we confess we place the highest confidence. We must refer our readers to the First Number of this Review, p. 239, where we have given a short notice of some recent observations by this celebrated physiologist upon the subject now before us, and will merely make two short quotations, which bear directly on the present question: "It was also evident," says Professor Baer, "that this corpus luteum was nothing else than the mucous membrane lining the Graafian vesicle, which, on account of its rapid growth, was puckered into rugæ;" in another place he says, "I observed, upon examining the body (viz. of a female who had drowned herself on the day after the occurrence of sexual intercourse,) a very turgid vesicle, and, on cutting through it, found the internal membrane, which resembles a mucous membrane, separated from the external one, evidently thickened, somewhat corrugated, and yellower than in the unimpregnated condition. The corrugation was, perhaps, produced in making the section; but this could not have taken place without previous detachment, because this inner membrane, before impregnation, is very firmly attached, throughout its whole extent, to the outer covering. Having frequently observed the lining of the Graafian vesicle in animals, thickened, and more or less detached from the external coat, before the vesicle had emptied itself, I had no doubt but that the growth of this mucous membrane precedes the opening of the vesicle, and that its opening, as also the discharge of the ovum, are effected by these means." We have made this last quotation from Professor Baer's paper, to show under what peculiar and unusual advantages (as respects the shortness of the internal after conception,) these interesting researches were made, opportunities which, without wishing to make the slightest approach to an invidious remark, we may say were not enjoyed by the author. We cannot admit the correctness of the assertion that the Graafian vesicle is lined with a serous membrane, nor do we know on what grounds the author has given it this character.

The description of the changes observable in the external form and appearance of the ovary are very excellent; it is true they are not superior to Meckel's, which is remarkably concise and comprehensive, but as this is not within the reach of every English reader, we quote our author on this subject:

"If we examine the ovaries of a pregnant woman, especially if her conception has been recent, we observe that the one which has supplied the germ differs in several remarkable particulars from its fellow of the opposite side: it strikes the eye at once as being larger, rounder, and more vascular; to the touch it feels fuller and softer: we perceive, further, that this increase of size of the one is not so much the result of an increased developement of the whole substance or body of the organ, as of the addition to it, at one part, of a tumour, projecting more or less from its natural outline, as we find in the eye, where the circumference of the cornea projects from the outline of the globe, the segment of a smaller circle being superimposed on that of a greater." (P. 220.)

Dr. Montgomery's description of the changes which the corpus luteum itself undergoes, as pregnancy gradually advances, is equally excellent: we can only quote a part of it:

"Its centre exhibits either a cavity or a radiated or branching white line, according to the period at which the examination is made; if within the first three or four months after conception, we shall, I believe, always find the cavity still existing, and of such a size as to be capable of containing a grain of wheat at least, and very often of much greater dimensions; this cavity is surrounded by a strong white cyst, (the inner coat of the Graafian vesicle), and, as gestation proceeds, the opposite parts of this cyst approximate, and at length close together, by which the cavity is completely obliterated, and in its place there remains an irregular white line, whose form is best expressed by calling it radiated or stelliform." (P. 226.)

We have not only the authority of Baer for stating that, during the early periods of gestation, the cavity of the corpus luteum is not "surrounded by a strong white cyst, (the inner coat of the Graafian vesicle)," but also that of Meckel, who expressly states that the corpus luteum, on close inspection, appears to consist of minute lobuli. It has appeared to us, since we were aware of Professor Baer's observations, that these lobuli were evidently formed by the corrugation of the lining membrane, above alluded to; and we have every reason to suppose that the "strong white cyst," as described by Dr. Montgomery, is not visible until a more advanced period; the drawings which he has given are, with one anormal exception, all of corpora lutea at a later period, which is proved by the external opening of the cavity having disappeared in every instance, showing that the corpus luteum had already undergone considerable changes, both as to size, structure, and induration.

In speaking of the discordant opinions which at one time prevailed as to the number of corpora lutea not being the same with that of the young produced, Dr. Montgomery has made some sound and very correct remarks:

"The presence of a corpus luteum does not prove that a woman has *borne a child*, although it would be a decided proof that she had been impregnated and had conceived; because it is quite obvious that the ovum, after its vivification, may be, from a great variety of causes, blighted and destroyed long before the fœtus has acquired any distinct form. It may have been converted into a mole or hydatids. Thus, however paradoxical it may at first sight appear, it is nevertheless obviously true, that a woman may conceive and yet not become truly with child,—but the converse will not hold good. I believe no one ever found a fœtus in utero without a corpus luteum in the ovary, and that the truth of Haller's corollary, 'Nullus unquam conceptus est absque corpore luteo,' remains undisputed." (P. 231.)

On this subject we must look upon Meckel as one of the highest authorities, he having examined the ovaries of no less than 200 animals of the class mammalia, and he always found that the number of corpora lutea corresponded exactly with that of the young produced; we may also add the names of Hunter, Blumenbach, and many others, in confirmation. Meckel has also declared his full conviction that a genuine corpus luteum was never found without the pre-occurrence of effectual sexual intercourse. Dr. Montgomery advocates the same opinion: "I wish to declare," says he, "that I never, in any one instance, saw the corpus luteum having the characters here described as belonging to it, except in females who had previously been impregnated, and my firm conviction is that such a corpus luteum was never found in a virgin animal." (P. 233.) He has entered fully into the consideration of this important point, and has collected much interesting literature to confirm the opinions which we have just stated, and which, we believe, are generally adopted by those who have at all examined the subject. We close our observations on the corpus luteum, by quoting a short but remarkably clear summary of the characters of those virgin corpora lutea, as they have been incorrectly termed:—

"1. There is no prominence or enlargement of the ovary over them.

"2. The external cicatrix is almost always wanting.

"3. There are often several of them found in both ovaries, especially in subjects who have died of tubercular disease, such as phthisis, in which case they appear to be merely depositions of tubercle, and are frequently without any discoverable connexion with the Graafian vesicles.

"4. They present no trace whatever of vessels in their substance, of which they are, in fact, entirely destitute, and of course cannot be injected.

"5. Their texture is sometimes so infirm that it seems to be merely the remains of a coagulum, and at others appears fibro-cellular, like that of the internal structure of the ovary, but never presents the soft rich lobulated, and regularly glandular appearance which Hunter meant to express when he described them as 'tender and friable, like glandular flesh.'

"6. In form they are often triangular, or square, or of some figure bounded by straight lines.

"7. They never present either the central cavity, or the radiated or stelliform white line, which results from its closure." (P. 245.)

Dr. Montgomery's article on the Period of Human Gestation is one of great interest, and demands an attentive consideration. We may truly affirm that, in no subject of such daily occurrence, and the determination of which is of so much importance, has there prevailed such discrepancy of opinion as in determining the duration of human pregnancy. When we call to mind the difficulties by which it is naturally surrounded, from the uncertainty of the date by which the female usually reckons, and the conflicting interests which, in questions of this sort, but too frequently embarrass and obscure the subject with every species of wilful manœuvring, we view the author's important observations with much interest, as tending to advance us a step further in this difficult investigation; and will endeavour, as far as lies in our power, to add such facts in our possession as may tend to confirm those views which have appeared to us nearest to the truth.

The chief points to be considered are two: firstly, the "natural period of gestation in women;" and secondly, the possibility of, and facts connected with, its protraction. We agree with the author that the natural term of pregnancy is 280 days, or forty weeks; and, in order to prevent the confusion which arises from considering this to be nine calendar months, (which is positively incorrect,) we have always been in the habit of reckoning by the lunar months, by which means the period of gestation will be ten months instead of nine.

"Calculations based," says Dr. Montgomery, "exclusively on the cessation of the catamenia must, necessarily, be defective in affording us any thing like precise information as to the exact period of human gestation: first, because conception may occur at any time between the termination of one menstrual appearance and the time of its expected return; ... secondly, there may be one or more monthly appearances after conception. ... My own observations lead me to the conclusion that conception occurs, in the great majority of instances, within the first week after the menstrual discharge. ... In some cases, and these by no means few, conception occurs immediately before the expected return of the menses; so that, of two women who may have menstruated on the same day, and conceived before the next return, one may complete her full term of gestation three, or even three and a half, weeks before the other; and hence a very common mode of calculation among women themselves is to reckon forty-two weeks from the last menstruation, or forty weeks from the middle day of the interval; and I think it is reasonable to believe that it was the adoption of this mode of calculation which induced the

Romans to allow, as the period of gestation, ten lunar months, amounting to 295 days, or forty-two weeks and one day; and the same period is also assigned by Harvey, dating from the last menstruation; when, says he, 'ten revolutions of the moone being expired, they are delivered, and reap the fruit of their wombe.'" (P. 256.)

It is the variations of the interval between the last appearance of the catamenia and the occurrence of conception, which, in our opinion, has been the fruitful source of uncertainty, and which have given rise to those extravagant, and but too frequently interested views, which have tended so much to perplex this difficult subject. The observations of Tessier on the duration of gestation in animals are highly interesting, but we cannot admit the fairness of applying these facts to the human female. The peculiarity of menstruation alone at once puts her under such essentially different circumstances, that it is impossible to reason correctly from the data alluded to. The occurrence of periodical excitement in the uterine system, which constitutes the function of menstruation, exerts a powerful influence on the phenomena of gestation; and it is a well-known fact, that, where premature expulsion has been induced by causes depending on the general condition of the system, that it usually occurs at what, in the unimpregnated state, would have been a menstrual period. On similar grounds, we have been for some years in the habit of explaining the usual termination of pregnancy at the fortieth week to result from the recurrence of a menstrual period at a time, during pregnancy, when the uterus, from its distention and weight of contents, is no longer able to bear that increase of irritability which accompanies these periods, without being excited to throw off the ovum. We offer this attempt at explanation with all deference, being but too aware that it still requires further confirmation. We should scarcely have ventured to proffer this view of the subject, had not the last-quoted observations of the author appeared to be grounded on a somewhat similar mode of reasoning. We can thus (at least to a certain extent,) easily account for the apparent over-term pregnancies which we every now and then hear of; the interval of time between the last appearance of the catamenia and the occurrence of conception being the chief cause of the variation. An *apparent* protraction of gestation may be by this means produced, as we have already shown in our last quotation from the author; but we conceive that gestation may be also *really* prolonged, under certain circumstances, by the same cause. Supposing two women (in whom the catamenia have regularly appeared every four weeks,) menstruate on the 1st of January: A conceives on the 2d, but B not until the 20th; the 7th of October following would, in the unimpregnated state, have been the tenth occurrence of the menstrual period from the 1st of January. A, being within two days of the forty weeks, or ten months, will not pass over this period, but will be confined on the 7th of October; whereas B, who has only gone 260 days, and is therefore still twenty days short of her full time, will, in all probability, pass by this period without labour coming on, and not be confined till about the 4th of November,—viz. the next menstrual period; and, as most women reckon from the last appearance of the catamenia, she will imagine that she has gone a month beyond the time, whereas she has only gone eight days.

"The weight of authority," says Dr. Montgomery, "is altogether on the side of those who believe in the occasional protraction of gestation." . . . "Many of these have, in confirmation of their opinions, related the cases on which their conviction was grounded, and which of course had fully satisfied their minds; and I cannot believe it possible that all of these writers could have been mistaken in a mere matter of fact or observation, and that none of the cases which they have put on record were really instances of gestation prolonged beyond forty weeks. At the same time I must add, that the cases which appear to me to carry with them the fullest demonstration of their truth, are those in which the ordinary term was not exceeded by more than three or four weeks." (P. 271.)

With this view of the subject we fully agree, and must express great doubts of cases which are said to have extended beyond four weeks over the time, even when attempted to be explained in the manner above mentioned. In looking over a list of cases which we made some years ago, where the duration of pregnancy had been determined from the last catamenial period, we find that, of 111 women, 57 went beyond, and 52 within, the term of 280 days from the last menstruation: in two instances labour came on exactly at 280 days from the above period. Reasoning merely from this means of reckoning, we should be inclined to infer that 280 days was scarcely the full term of pregnancy; whereas, if we compare the duration of pregnancy in those who have reckoned from other and surer data, we shall find a very different result. Thus, in three cases within our personal knowledge, where intercourse took place but once, the first woman went 260 days; the second, 264 days; and the third, 276 days. Again, of four patients who dated conception from their own peculiar feelings, the first went 272, the second 275, the third 277, and the fourth 291 days; but of the last we have some doubts. Of four patients who had no other date to reckon by except that of their marriage, the first went 256, the second 273, the third 285, and the fourth 288 days. Hence we may conclude that, where the data of conception are tolerably correct, the duration of pregnancy is rarely above the term of 280 days; and, if it be, that this will be capable of being explained in the manner we have just proposed.

In speaking of the Signs of Delivery, we must confine ourselves to a very few brief observations. The changes in the breasts are those to which the author has called our attention first.

"The presence of broken streaks, running in nearly concentric curved lines, of a shining white or sometimes pearly colour, most numerous on the lower part of the abdomen, and sometimes observed on the nates and upper part of the thighs, like the remains of numerous small cicatrices, the surface of which seems reticulated, or as if the texture of the skin had been frayed, is a sign of acknowledged value." . . . "It sometimes happens also, especially in young women of a full habit, that, when the breasts have been greatly and rapidly enlarged during pregnancy and after delivery, the skin covering them is in like manner injured, and silvery lines are formed, which never afterwards disappear." . . . "It is very important to know that these streaks may form on the skin of the breast *during pregnancy*, as well as after delivery; for otherwise we might be led into serious error, and conclude, in a case of first pregnancy, where they happened to be developed during gestation, that the woman has been delivered before. But I have now seen a sufficient number of instances to convince me that they unquestionably form, in some cases of first pregnancy, so early as the sixth month." (P. 296.)

Although the presence of these silvery streaks in the skin of the breast will afford a pretty clear proof that pregnancy has existed, still their absence is very far from proving the converse. We are convinced that not only pregnancy, but lactation, may take place without their being visible: on the other hand, as is the case with the white lines upon the abdominal parietes, they are capable of being produced by distention of the part from other causes besides those connected with pregnancy.

"The state of the os uteri, vagina, and external parts next claims our attention. By an examination per vaginam, we detect the enlarged state of the uterus, and its identity with the abdominal tumour; and at the same time we ascertain the condition of the os uteri, which, in a recently delivered woman, is found gaping open, so that two or three fingers might be introduced into it with ease: its margins are flabby and very much relaxed, and not unfrequently feel as if divided by several small fissures." (P. 304.) . . . "When the os uteri of a woman who has borne children is examined, its labia are in general found jagged or notched, and sometimes as if a portion had been torn, and remained separated from the rest. I attach great consideration to this state of the part, because it is not likely to be produced by the expulsion of any accidental formation from the cavity of the uterus, and I have never met with it except after childbirth; nor do I believe that it is ever the natural original condition of the uterine orifice." . . . "But the converse of this will not hold good; the unfissured state of the uterine orifice will not be sufficient proof against the former occurrence of childbirth; for a woman may have been delivered, even of a full-grown child, without the production of this change in the os uteri, or only in a trifling degree." (P. 298.)

The condition of the os uteri for some little time after labour is a subject well worthy of attention. To ascertain its precise state, and the changes which it gradually undergoes during the first fortnight after labour, we were induced, several years ago, to institute a series of examinations, in a number of patients, at the end of the first and second week after labour. We will quote two of these examinations, as they give a correct enumeration of these changes:—"*Oct.* 9. —— delivered seven days ago. Os uteri high up, its edges very soft and gaping, admitting the finger with ease; anterior lip longer than the posterior one. —*Oct.* 16. Os uteri deep in the centre of the pelvic cavity, round, somewhat harder; os uteri externum admits the point of the finger easily, but the os uteri internum does not."

Dr. Montgomery's observations apply to a much earlier period after labour, as is seen by the following quotation.

"If the examination happens to be made within a few hours after delivery, the patulous state of this orifice is such that its margins cannot be distinctly recognized, so that we feel at a loss to distinguish between it and the cavity of the vagina, of which it seems as if it were a continuation. This latter part also is greatly relaxed and dilated, in consequence of which its internal surface is rendered smooth; its natural rugæ being obliterated by the recent distension of its tissues." (P. 304.)

The author's observations on the size and development of the uterus at different intervals after labour are very correct; but we are surprised that he has paid no attention whatever to the size of the uterus, during the first forty-eight or sixty hours after labour; as we feel quite confident that the height of the fundus above the symphysis pubis will greatly assist in proving how much time has elapsed since delivery. The uterus does not continue to contract in size from the moment of expulsion, but in a very short time *increases* in size, becomes somewhat softer, and continues to increase for at least forty-eight hours: it now becomes harder and smaller, and undergoes that gradual diminution which has been described by authors. To render this subject more intelligible, we will give some admeasurements at different periods after labour, as they occurred in one among several cases. Immediately after birth, the fundus was felt three fingers' breadth below the umbilicus; in ten minutes afterwards, it was two fingers' breadth; in eight hours, it was one finger's breadth; and in twenty hours, level with the umbilicus. On the second day, it was one finger's breadth above, and the third day four fingers below, the umbilicus.

Our limits will not allow us to enter further into this interesting subject, for they have been already long since overstepped. Dr. Montgomery's remarks are highly instructive, and we recommend them to our readers.

The last article in the volume is on the "Spontaneous Amputation of the Fœtal Limbs in Utero," originally published in the Dublin Journal of Medical Science. We have only room to observe that Dr. M.'s view of the subject is by far the most rational which has yet been offered, and which we adopted as soon as we read his interesting paper. We have ourselves met with a remarkable case of amputation of the arm where the child was at the full term: she is now a girl of about seventeen. We were assured by the parents that the separated limb was scarcely, if at all, smaller than the other.

We here close a lengthened, and we trust impartial review of Dr. Montgomery's work, which we strongly recommend to our readers as by far the completest and best that exists on the subject of which it treats. We have unhesitatingly expressed our dissent wherever we felt reason to do so, and we feel convinced that the excellent author will not take in ill part the critical observations which we have thought fit to make. The remarks which we have ventured to make on the period of gestation have not been made without reflection; and we may also state that they have been furnished from very extensive sources: nevertheless, as before observed, we offer them as opinions which still require further investigation and proof. Besides its great intrinsic value, which recommends it alike to the student and practitioner, the work is interspersed with so much interesting illustration as to make it not less a source of amusement than of instruction. We have already spoken of the plates, which are admirable; and we may add, that the whole book is a good specimen of the superior "*getting-up*" of the present day.

---

